# Insights into the role of RNA m^6^A modification in the metabolic process and related diseases

**DOI:** 10.1016/j.gendis.2023.04.038

**Published:** 2023-07-04

**Authors:** Haiming Hu, Zhibin Li, Xia Xie, Qiushi Liao, Yiyang Hu, Chunli Gong, Nannan Gao, Huan Yang, Yufeng Xiao, Yang Chen

**Affiliations:** Department of Gastroenterology, Xinqiao Hospital, Army Medical University, Chongqing 400037, China

**Keywords:** Cancer, m^6^A-targeted therapy, Metabolic disease, Metabolism, N6-methyladenosine

## Abstract

According to the latest consensus, many traditional diseases are considered metabolic diseases, such as cancer, type 2 diabetes, obesity, and cardiovascular disease. Currently, metabolic diseases are increasingly prevalent because of the ever-improving living standards and have become the leading threat to human health. Multiple therapy methods have been applied to treat these diseases, which improves the quality of life of many patients, but the overall effect is still unsatisfactory. Therefore, intensive research on the metabolic process and the pathogenesis of metabolic diseases is imperative. N6-methyladenosine (m^6^A) is an important modification of eukaryotic RNAs. It is a critical regulator of gene expression that is involved in different cellular functions and physiological processes. Many studies have indicated that m^6^A modification regulates the development of many metabolic processes and metabolic diseases. In this review, we summarized recent studies on the role of m^6^A modification in different metabolic processes and metabolic diseases. Additionally, we highlighted the potential m^6^A-targeted therapy for metabolic diseases, expecting to facilitate m^6^A-targeted strategies in the treatment of metabolic diseases.

## Background

Normal cellular metabolism, including the metabolism of nucleic acids, amino acids, lipids, and carbohydrates, provides energy and materials for life activities. Metabolic progress is regulated by multiple metabolic enzymes, while the expression and activity of those enzymes are precisely regulated at different levels. Aberrant cellular metabolism is closely related to the occurrence and/or development of many diseases, such as hyperlipemia,^1^ obesity,^2^ hypoglycemia,^3^ diabetes,^4^ and cancer.^5^ Epigenetic modification is an important regulator of cellular metabolism processes by modifying the expression and/or activity of certain genes. Therefore, research on the relationship between epigenetic modification and metabolic regulation is of great significance for elucidating the mechanisms of these diseases and improving therapeutic strategies.

Abundant and wide studies have shown that epigenetic modification of RNAs exerts a crucial role in the regulation of cellular metabolic processes.^6^ In the mid-twentieth century, pseudouridine (Ψ) was clarified as the first modification on RNA,^7^ and to date, more than 170 different RNA modifications have been clarified, including methylation, 5′cap, and 3′polyadenylation. Methylation is the most common modification on RNA, mainly including pseudouridine, N1-methyladenosine (m1A), 2′-O-methylations (2′-O-Me), 5-methylcytosine (m5C), N6-methyladenosine (m^6^A), and N7-methylguanosine (m7G). In the 1970s, RNA m^6^A modification was first discovered in eukaryotes, but there was no breakthrough progress in correlational research until recent advances in m^6^A detection technology. In 2012, two research groups improved the m^6^A detection method called methylated RNA m^6^A immunoprecipitation sequencing (MeRIP-m^6^A-seq), which allows researchers to investigate RNA m^6^A modification much more easily.^8^^,^^9^ To date, numerous investigations have indicated that m^6^A modification plays an essential role in multiple cellular processes, including metabolic processes and related metabolic diseases.

The m^6^A modification requires active methyl compounds as donors that come from different metabolic pathways, while the mRNA of many metabolic enzymes can be modified by m^6^A modification. In this review, we summarized the classic processes of m^6^A modification and its role in the regulation of metabolic enzyme expression and highlighted the aberrant m^6^A levels in different metabolic diseases. Additionally, we briefly discussed the current research status of m^6^A-targeted therapy in the treatment of metabolic diseases, which indicated that RNA m6A methylation represents the potential target for the treatment of metabolic diseases.

## Overview of RNA m^6^A modification

As one of the most prevalent RNA modifications, m^6^A modification was first discovered in mouse L cell mRNA in the early 1970s.^10^ Subsequent studies also identified m^6^A modification in yeast.^11^ However, due to the limitation of the detection technique, the m^6^A modification could not be measured in individual transcripts for a long time. In recent years, the emergence of MeRIP-m^6^A-seq has provided an easier method to clarify the specific m^6^A modification on RNAs. On this basis, multiple studies have shown that m^6^A modification is a dynamic and highly conserved process.^12^ The m^6^A modification site mainly occurs near the starting position of the 3′ untranslated region (3′UTR) of mRNAs,^8^ while modification of the coding region sequence (CDS),^13^ 5′UTR,^14^ and noncoding RNA^15^^,^^16^ has also been reported. In this part, we will give an outline of the classic m^6^A modification.

**Figure 1:**
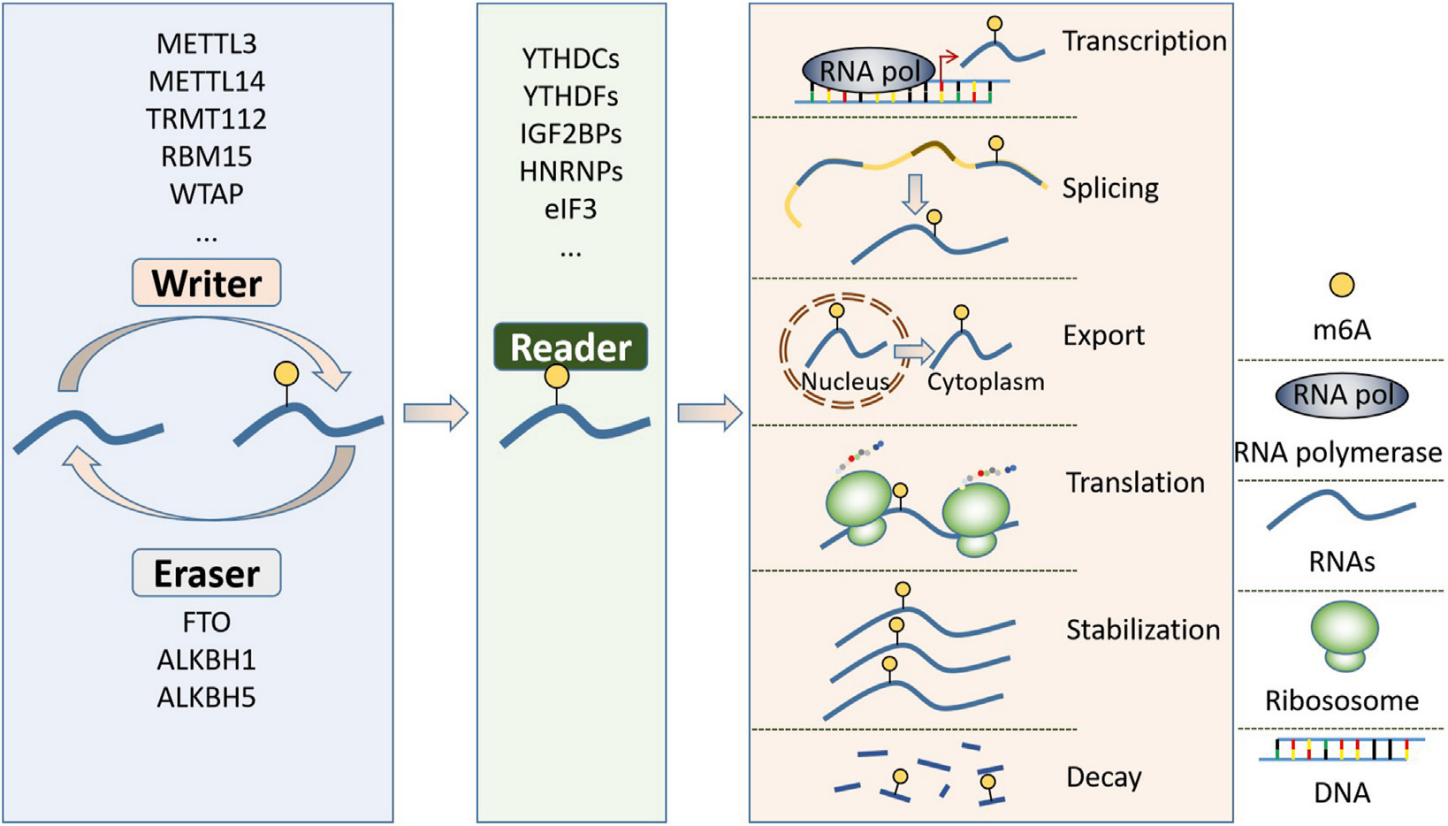
Overview of RNA m^6^A modification. The RNA m^6^A modification is dynamically catalyzed by methyltransferases, demethylases, and m^6^A binding proteins which are also called ‘writers’, ‘erasers’, and ‘readers’, respectively. The m^6^A writers mediate the methylation of the targets while the erasers perform a opposite role. The function of the readers is multitudinous that depending on the specific reader and target.

The classic m^6^A modification process is dynamically regulated by methyltransferases, demethylases, and m^6^A binding proteins, which are also called ‘writers’, ‘erasers’, and ‘readers’, respectively (Fig. 1 and Table 1). To date, the identified methyltransferases (writers) include methyltransferase-like 3 (METTL3), METTL14, METTL16, Wilms tumor 1-associated protein (WTAP), zinc finger CCCH-type containing 13 (ZC3H13), and RNA-binding motif protein 15/15 B (RBM15/15 B). METTL3 is the core subunit that binds to S-adenosyl methionine (SAM) directly. METTL14 interacts with METTL3 and binds to RNA, thus promoting methyl group transfer to adenosine.^17^^,^^18^ WTAP, an adaptor of METTL3, promotes the translocation of the METTL3-METTL14 heterodimer to the nuclear speckle.^19^ Usually, they can catalyze the transfer of methyl from active methyl compounds to specific substrates directly or indirectly. The demethylases (erasers) include Fat mass- and obesity-associated gene (FTO), AlkB homolog 1 (ALKBH1), and ALKBH5, which eliminate methyl groups from the m^6^A modification sites. In 2012, Jia et al first reported that FTO is an m^6^A demethylase.^20^ Zheng et al found that ALKBH5 also exhibits m^6^A demethylation activity.^21^ ALKBH1 is the latest demethylase identified by Wu et al in 2016.^22^ Research has indicated that FTO has a higher affinity for N6,2-O-dimethyladenosine (m^6^A_m_) than for m^6^A, while ALKBH5 has no m^6^A_m_ elimination activity.^23^ The m^6^A binding proteins (readers) mainly contain eukaryotic initiation factor 3 (eIF3),^14^ YT521-B homology (YTH) domain-containing proteins,^24^^,^^25^ insulin-like growth factor 2 mRNA binding proteins (IGF2BPs),^26^ and heterogeneous nuclear ribonucleoprotein (HNRNPs) family,^27^ which specifically recognize and bind to m^6^A-modified sites and have a distinct function on RNA processing.

**Table 1:** The identified m^6^A regulators.

Type	Name	Full name	Reference
Writer	METTL3	Methyltransferase-like 3	17
	METTL5	Methyltransferase-like 5	215
	METTL14	Methyltransferase-like 14	18
	METTL16	Methyltransferase-like 16	208
	WTAP	Wilms tumor 1- associated protein	19
	VIRMA (KIAA1429)	Vir-like m6A methyltransferase associated	209,210
	RBM15	RNA binding motif protein 15	211
	RBM15B	RNA binding motif protein 15 B	211
	ZC3H13	Zinc Finger CCCH-Type Containing 13	212
	HAKAI	HAKAI	213
	ZCCHC4	ZCCHC4	214
	TRMT112	tRNA methyltransferase activator subunit 11-2	215
Eraser	FTO	Fat mass- and obesity-associated gene	216
	ALKBH1	AlkB homolog 1	22
	ALKBH5	AlkB homolog 5	217
Reader	YTHDC1	YTH domain containing 1	33
	YTHDC2	YTH domain containing 2	218
	YTHDF1	YTH N6-methyladenosine RNA binding protein 1	219
	YTHDF2	YTH N6-methyladenosine RNA binding protein 2	220
	YTHDF3	YTH N6-methyladenosine RNA binding protein 3	24
	Mrb1	Mitochondrial RNA-binding complex 1	221
	eIF3	Eukaryotic initiation factor 3	14,222
	HNRNPA2B1	Heterogeneous nuclear ribonucleoprotein A2B1	223,224
	HNRNPC/G	Heterogeneous nuclear ribonucleoprotein C/G	225
	IGF2BP1/2/3	Insulin like growth factor 2 mRNA binding protein 1/2/3	83
	FMRP	Fragile *X* mental retardation protein	226
	Ribosome	Ribosome	38
	ELAVL1	ELAV Like RNA Binding Protein 1	227
	LRPPRC	Leucine rich pentatricopeptide repeat containing	228
	PRRC2A	Proline rich coiled-coil 2 A	229

## The m^6^A modification in RNA processing

**Figure 2:**
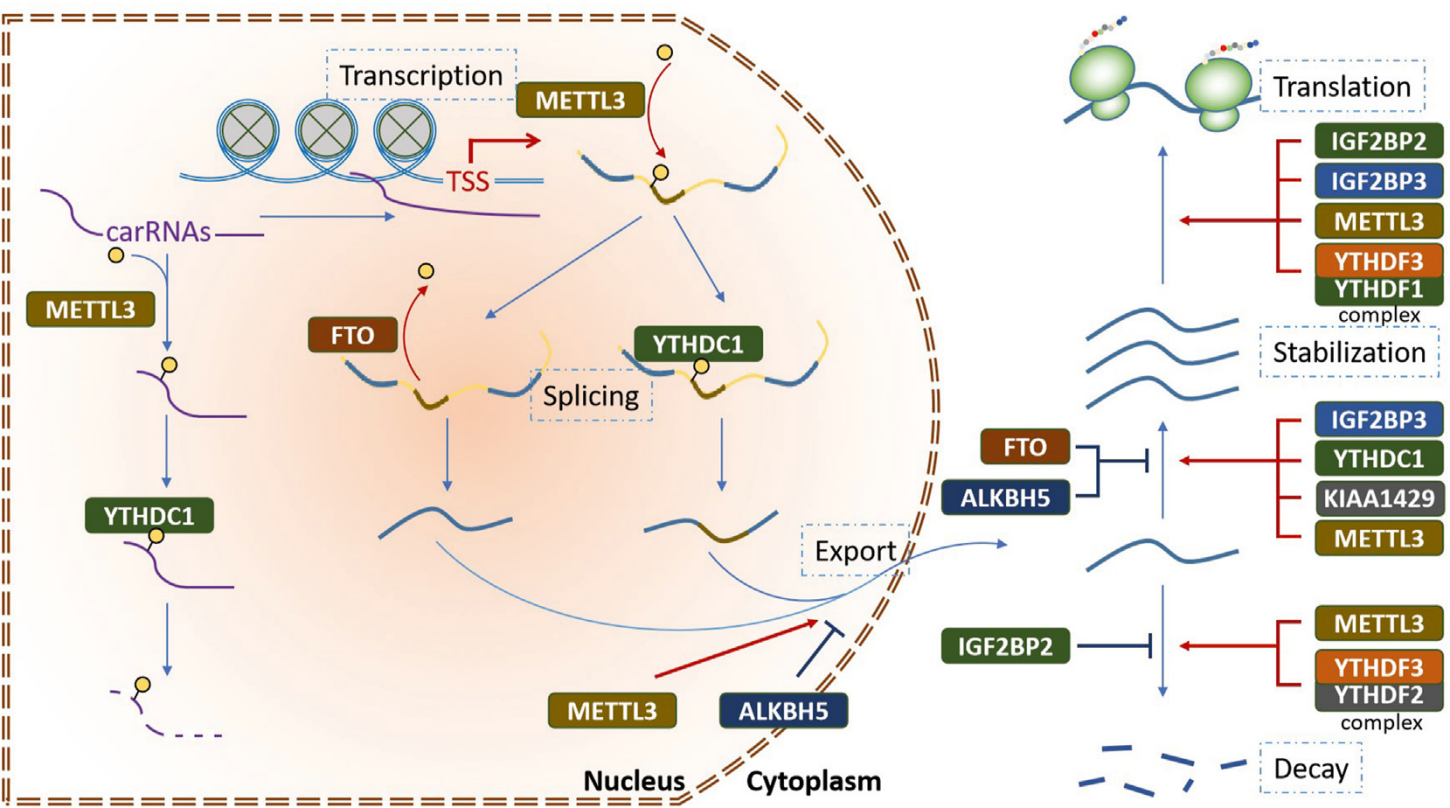
The functions of m^6^A modification in RNA processing. The m^6^A modification regulates RNA processing at different levels including chromatin accessibility, DNA transcription and splicing, RNA nuclear-plasma transport, stability, and translation.

RNA mainly acts as a storage and transmission media of life information. RNA processing is an enzyme-mediated process that has been proven to be regulated by m^6^A modification. In this section, we summarized the m^6^A-mediated regulation of RNA processing (Fig. 2 and Table 2).

**Table 2:** The m^6^A modification in RNA processing.

m^6^A regulator	Target	Mechanism	Function	Reference
METTL3	CCAAT-box containing genes	Colocalized with CEBPZ to CCAAT-box	Maintenance of the leukaemic state	29
	Per2, Arntl	↑ mRNA maturation and transport	Maintain circadian period	36
	Pri-miRNAs	↑ The binding of DGCR8 to Pri-miRNAs	Promotes the maturation of miRNAs	16
	STRA8	Regulates expression and alternative splicing	Maintain male fertility and spermatogenesis	35
METTL14	PERP	↓ mRNA stability	Promotes pancreatic cancer	230
METTL16	MAT2A	alternative splicing	mRNA splicing	208
WTAP	METTL3, METTL14	↑ Nuclear speckle translocation of the heterodimer	Regulates transcription and alternative splicing	19
FTO	MYC, CEBPA	↑ Stabilitby of MYC/CEBPA	↑ MYC/CEBPA transcripts	48
ALKBH5	ASF/SF2	Alters mRNA process, export and metabolism	Regulates mRNA process, export and metabolism	21
YTHDF1	MTCH2	↑ MTCH2 translation	↑ mRNA translation	77
	HK2	↑ mRNA stability	↑ mRNA stability	95
	YAP	Recruits eIF3b to translation initiation complex	↑ mRNA translation	219
YTHDF2	CDK2, CCNA2	↓ mRNA stability	↓ mRNA stability	65,203
	OCT4	↑ translation	↑ liver cancer stemness	39
YTHDF3	YTHDF1, YTHDF2	↑ YTHDF1translation, YTHDF2 degradation	Regulates translation and degradation	24
YTHDC1	Pre-mRNA	The nuclear speckle localization of SRSF3↑ or SRSF10↓	↑ mRNA splicing	33
	miR-30 d	↓ Pri-miR-30 d stability	↑ Aerobic glycolysis	108
YTHDC2	Spermatogenesis related genes	Recruits the CCR4-NOT deadenylase complex	Maintains spermatogenesis	218
	SREBPC1, ACC1, FASN	↓ mRNA stability	↓ Liver steatosis	79
IGF2BP 1/2/3	MYC	↑ mRNA stability	↑ Aerobic glycolysis	83

Note: ↑ means upregulation and ↓ means downregulation.

Multiple studies have indicated that the processing of mRNA is regulated by m^6^A modification.^23^^,^^28^ Firstly, m^6^A modification regulates the transcription of target genes. Research has shown that METTL3 accumulates at the transcriptional start sites of targeted genes where the CAATT-box binding protein CEBPZ is present and induces m^6^A modification of associated mRNA within the coding region transcript, which leads to enhanced translation.^29^ In mouse embryonic stem cells, METTL3 and YTH domain containing 1 (YTHDC1) also regulate chromatin accessibility by modifying the m^6^A level of chromosome-associated regulatory RNAs (carRNAs), which leads to the altered transcriptional activity of multiple genes.^15^

Secondly, m^6^A modification is involved in the maturation of RNA transcripts. In HEK293T cells, FTO can bind to the intronic regions of pre-mRNAs to regulate their splicing, and FTO knockout increases exon skipping events.^30^ FTO depletion also increases the inclusion of target exons by enriching m^6^A modification in their 5′ and 3′ splice sites, which inhibits the differentiation of adipocytes.^31^ Numerous studies have indicated that YTHDC1 regulates pre-mRNA splicing by recruiting splicing factors to its targets in many cell types.^32^, ^33^, ^34^ METTL3 knockout in mice alters the expression and alternative splicing of spermatogenesis-related genes, which leads to reduced spermatogonial differentiation and meiosis initiation.^35^ In addition, depletion of METTL3 or ALKBH5 can inhibit or enhance mRNA export, respectively.^21^^,^^36^ The microprocessor complex subunit DGCR8 is a key factor in pri-miRNA cleavage into pre-miRNAs. In breast cancer cells, METTL3-mediated methylation of pri-miRNAs increases the binding and processing of DGCR8 on pri-miRNAs, which promotes the maturation of target miRNA.^16^

Thirdly, protein translation is regulated by m^6^A modification. Research indicated that METTL3-eIF3h complex tethers to the m^6^A modified stop codon of targeted mRNAs to promote their translation.^37^ YTHDF1 binds to m^6^A-modified mRNAs to promote their translation by interacting with ribosomes and initiation factors, while YTHDF2 promotes mRNA decay.^38^ Interestingly, YTHDF3 enhances or suppresses mRNA translation depending on binding to YTHDF1 or YTHDF2.^24^ In liver cancer, YTHDF2 increases the m^6^A level in the 5′UTR of OCT4 mRNA leading to enhanced protein translation of OCT4, and mutation in the corresponding m^6^A modification site decreases OCT4 expression.^39^ Moreover, Li et al also reported that YTHDF3 promotes the translation of its targets by combining with YTHDF1.^40^

Finally, m^6^A modification is a vital regulator of mRNA stability. Studies have indicated that METTL3 directly reduces mRNA stability in a variety of cells.^41^^,^^42^ ALKBH5 shortens the half-life of CYR61 mRNA and decreased its expression.^43^ YTHDF2 could recognize the m^6^A-modified RNAs via its C-terminal domain, and then direct the RNAs to the CCR4-NOT degradation machinery via the N-terminal domain.^44^^,^^45^ However, the increased m^6^A modification stabilizes specific mRNAs, such as glucose transporter 1 (GLUT1) and c-Myc, under hypoxic exposure.^46^ In myeloid leukemia, nuclear YTHDC1-m^6^A condensates (nYACs) enable YTHDC1 to protect m^6^A-modified mRNAs from degradation and maintain cell survival and an undifferentiated state.^47^ Additionally, inhibition of FTO activity by R-2-hydroxyglutarate (R-2HG) increases global m^6^A modification, which reduces the stability of MYC/CEBPA transcripts in leukemia cells.^48^

In summary, m^6^A modification regulates RNA metabolism via multiple pathways, and the outcomes of m^6^A-modified RNAs are distinct depending on tissue and cell type.

## The function of m^6^A modification in cellular metabolism

All cellular processes require metabolism to ensure continual energy and material supply, which are regulated by various metabolic enzymes. Multiple investigations have indicated that m^6^A modification is a master regulator of metabolic processes by regulating the expression and/or activity of these enzymes. In this section, we summarized the function of m^6^A modification in the regulation of different metabolic processes, such as lipids, carbohydrates, and amino acids (Table 3).

**Table 3:** The function of m^6^A regulators in different metabolic processes.

Type	m6A regulator	Target	Mechanism	Function	Reference
**Lipid metabolism**	METTL3	↑ TRAF6	↑ mRNA export	↓ LCFAs absorption	58
		↑ JAK1	↓ mRNA stability	↓ BMSC adipogenic differentiation	55
		↑ FASN	↑ mRNA expression	↑ Fatty acid synthesis	60
		↑ ERRγ	↑ mRNA maturation	↑ β-oxidation	61
		↓ CCND1	↓ mRNA stability	↓ Lipid droplet accumulation	53
	FTO	↑ RUNX1T1	↑ mRNA splicing	↑ Lipogenesis	31
		↑ PPARγ	↓ mRNA stability	↑ Adipocyte differentiation	64
		↑ CCNA2, CDK2	↑ mRNA stability	↑ Adipogenesis	65
		↑ JAK2	↑ mRNA stability	↑ Adipogenesis	66
		↑ FASN	↑ mRNA stability	↑ Lipid accumulation	68
		↑ SREBP1c	↑ mRNA stability	↑ Adipogenesis and lipid accumulation	69
		↓ AMPK, PPARβ/δ	↓ mRNA stability	↓ Lipid oxidation	69
		↑ CD36	↑ mRNA stability	↑ Inflammation of LHD	74
		↑ C/EBPβ	↑ mRNA stability	↑ Preadipocyte differentiation	69
	ALKBH5	↑ CES2	↑ mRNA stability	↓ Lipid accumulation	75
	YTHDF1	↑ MTCH2	↑ mRNA stability	↑ Adipogenesis	77
		↑ PNPLA2	↑ mRNA stability	↓ Lipid accumulation	231
		↓ HSD17B11	↓ mRNA stability	↓ Lipid droplets formation	76
	YTHDF2	↓ CCNA2, CDK2	↓ mRNA stability	↓ Adipogenesis	65
		↓ JAK2	↓ mRNA stability	↓ Adipogenesis	66
		↓ ATG5, ATG7	↓ mRNA stability	↓ Adipogenesis	71
		↓ FIP200	↓ mRNA stability	↓ Autophagy	78
		↓ PPARα	↓ mRNA stability	↓ Adipogenesis	81
	YTHDC2	↓ SREBP1c, FASN, ACC1	↑ mRNA stability	↓ Triglyceride deposition	79
	HNRNP A2B1	↑ ACLY, ACC1	↑ mRNA stability	↑ Lipid accumulation	82
**Carbohydrate metabolism**	METTL3	↑ HK2, GLUT1	↑ mRNA stability	↑ Aerobic glycolysis	96
		↑ FASN	↑ mRNA stability	↓ Insulin sensitivity	60
		↑ GLUT1	↑ mRNA translation	↑ Glucose uptake and lactate production	97
		↑ HK2	↑ mRNA stability	↑ Aerobic glycolysis	95
		↑ MarfA	↑ mRNA stability	↑ Maturation of β cells	88
	METTL14	↑ Ins1, Ins2, CPE	↑ mRNA translation	↑ Insulin secretion	89
		↓ BPTF	↓ mRNA stability	↑ Aerobic glycolysis	99
	WTAP	↑ ENO1	↑ mRNA stability	↑ Aerobic glycolysis	93
		↑ HK2	↑ mRNA stability	↑ Aerobic glycolysis	94
	FTO	↑ FOXO1, G6PC, DGAT2	↑ mRNA stability	↑ Hyperglycemia	91
		↓ APOE	↓ mRNA stability	↑ Aerobic glycolysis	102
		↑ PDK1	↑ mRNA stability	↑ Aerobic glycolysis	105
		↓ MYC	↓ mRNA translation	↓ Aerobic glycolysis	104
		↑ FOXO1	↑ mRNA translation	↑ Gluconeogenesis	103
		↑ G6PC	↑ mRNA transcription	↑ Gluconeogenesis	100
		↑ G6P	↑ mRNA transcription	↑ Gluconeogenesis	101
	ALKBH5	↓ CK2α, GLUT, HK1	↓ mRNA stability	↓ Aerobic glycolysis	106
	YTHDF1	↑ PDK4	↑ mRNA stability	↑ Aerobic glycolysis	107
	YTHDC1	↓ miR-30 d	↑ RNA degradation	↓ Aerobic glycolysis	108
	KIAA 1429	↑ GLUT1	↑ mRNA stability	↑ Aerobic glycolysis	98
	IGF2BPs	↑ MYC	↑ mRNA stability	↑ Aerobic glycolysis	83
	IGF2BP2	↑ MYC	↑ mRNA stability	↑ Aerobic glycolysis	140
		↑ PDX1	↑ mRNA translation	↑ Insulin secretion	90
	IGF2BP3	↑ PDK4	↑ mRNA stability	↑ Aerobic glycolysis	107
**Amino acids metabolism**	METTL16	↑ BCAT1, BCAT2	↑mRNA stability	↓BCAA	111
	YTHDF1	↑ GLS1	↑ mRNA translation	↑Glutaminase	113
**Mitochondrial function**	METTL3	↓ PGC-α	↑ mRNA degradation	↓ ATP generation	115
	FTO	↑ PGC-α	↑ Ddit4 mRNA	↑ ATP generation	116
		↑ PGC-α	↑ mRORC1	↑ ATP generation	119
	YTHDF2	↓ PGC-α	↑ mRNA degradation	↓ ATP generation	115
	IGF2BP2	↑ Bmi1	↓ mRNA degradation	↓ ATP generation	117

Note: ↑ means upregulation and ↓ means downregulation.

## The m^6^A modification in lipid metabolism regulation

**Figure 3:**
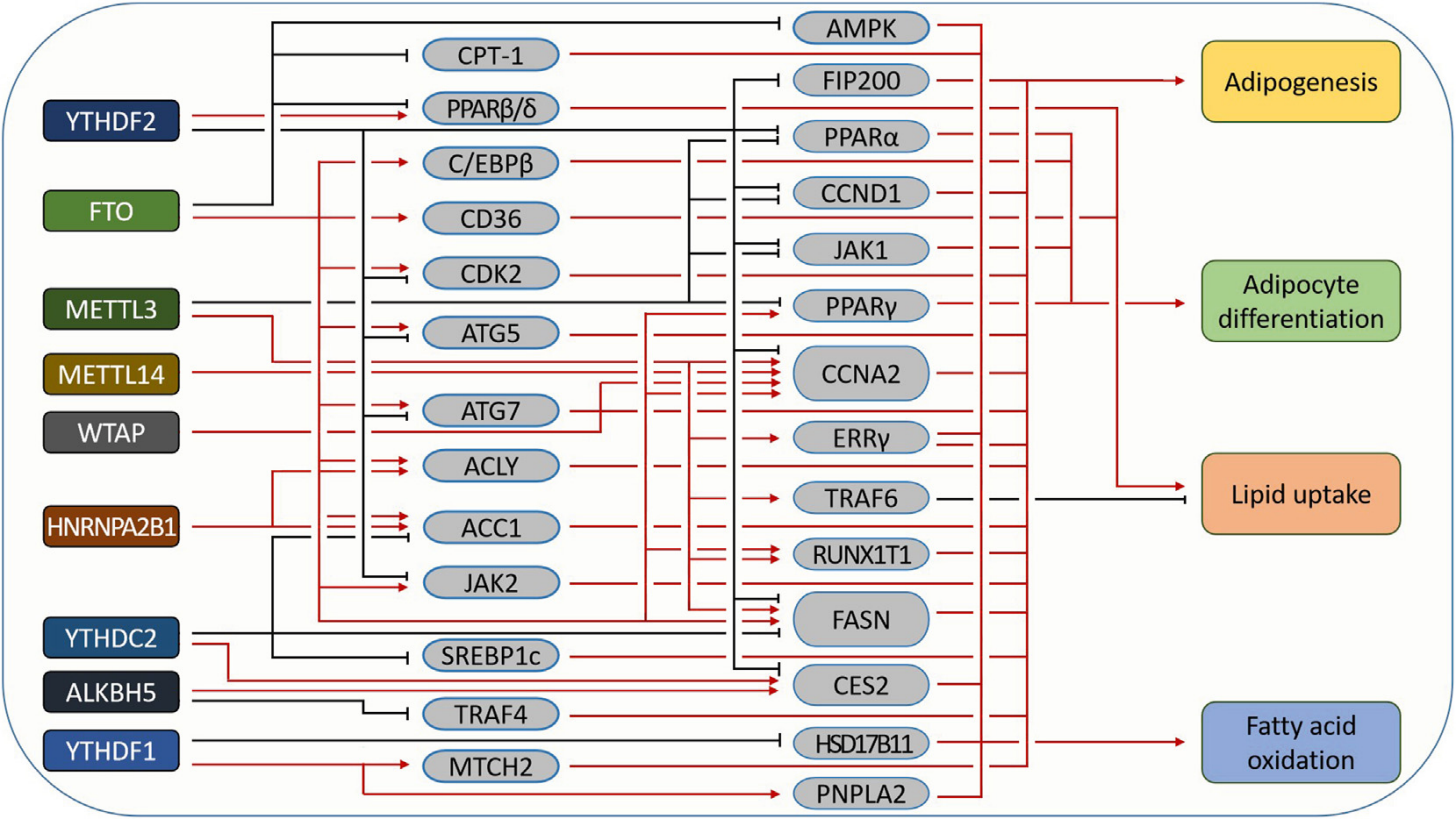
The function of m^6^A modification in lipid metabolism. Many m^6^A regulators are involved in the regulation of multiple lipid metabolism-related genes at different levels. They play an important role in the regulation of adipogenesis, lipid uptake, fatty acid oxidation, adipocyte differentiation, *etc*.

Lipids, containing fatty acids, phospholipids, cholesterol, and related derivatives, are important materials of energy, structural components of membranes, and signaling molecules. The lipids are mainly stored in adipocytes derived from mesenchymal stem cells (MSCs).^49^ The intracellular lipids come principally from extracellular uptake and intracellular *de novo* synthesis. Many studies have indicated that some lipid metabolism-related genes are regulated by m^6^A modification (Fig. 3).

Yadav et al reported that IME4 (m^6^A methyltransferase in yeast) plays an essential role in the regulation of peroxisomal biogenesis, long-chain fatty acyl-CoA synthetase, and mitochondrial function.^50^^,^^51^^,^^56^ In 3T3-L1 cells, ZFP127 depletion promotes METTL3 expression and then increases the m^6^A level and suppresses YTHDF2-mediated degradation of cyclin D1 mRNA, leading to inhibited adipogenesis.^52^ Wang et al found that METTL3 increases m^6^A modification level and inhibits adipogenesis in porcine adipocytes.^53^ Yao et al found that METTL3 knockout reduces the m^6^A level of Janus kinase 1 (JAK1) mRNA, leading to increased mRNA stability and expression of JAK1, and thus promoting bone marrow stromal cell (BMSC) adipogenic differentiation.^54^ In brown adipose tissue, METTL3 deletion decreases the m^6^A modification and expression of the PR domain containing 16 (PRDM16), uncoupling protein 1 (UCP-1), and peroxisome proliferator-activated receptor gamma (PPARG) and thereby promotes high-fat diet-induced obesity.^55^ METTL3 can promote ox-LDL-mediated inflammation by activating the signal transducer and activator of transcription 1 (STAT1).^57^ METTL3 knockout *in vitro* exerts anti-malabsorption of long-chain fatty acid (LCFA) activity by decreasing the expression of TNF receptor-associated factor 6 (TRAF6), leading to suppression of the NF-κB and MAPK signaling pathways, thereby suppressing inflammation and increasing the absorption of LCFAs.^58^ However, in 3T3L1 cells, METTL3 promotes adipogenesis by promoting cell cycle transition.^59^ In high-fat diet-fed mice, METTL3 knockdown reduces the m^6^A mRNA level of fatty acid synthase (FASN), leading to a decreased fatty acid abundance.^60^ In HepG2/ADR cells, Chen et al found that METTL3 can up-regulate m^6^A and trigger splicing of precursor mRNA of estrogen-related receptor γ (ERRγ), which increases fatty acid oxidation (FAO) in chemoresistant cells through regulation of the rate-limiting enzyme carnitine palmitoyltransferase 1 B (CPT1B).^61^ These results suggested that the function of the m^6^A ‘writers’ is species- and cell-dependent and requires further investigation.

As an m^6^A ‘eraser’, FTO was first identified as a regulator of human body mass, and studies also found that adipose tissue is significantly reduced in FTO-deficient mice compared with wild-type mice.^62^^,^^63^ A follow-up study found that FTO promotes BMSC differentiation into adipocytes by increasing PPARγ expression.^64^ Depletion of FTO inhibits adipogenesis by decreasing the expression of cyclin-dependent kinase 2 (CDK2) and cyclin A2 (CCNA2), leading to delayed cell cycle entry of adipogenesis.^65^ In porcine and mouse preadipocytes, FTO deficiency attenuates the transcription of C/EBPβ by suppressing JAK2 expression and STAT3 phosphorylation.^66^ In HepG2 cells, FTO promotes triglyceride deposition by decreasing m^6^A level.^67^ FTO knockdown increases the m^6^A levels of FASN mRNA, suppressing FASN expression and inhibiting lipid accumulation through an m^6^A-dependent manner.^68^ In the liver, FTO overexpression promotes lipogenesis and lipid droplet accumulation, but decreases CPT-1-mediated FAO through sterol regulatory element-binding protein-1c (SREBP1c), leading to increased lipid storage and nonalcoholic fatty liver diseases (NAFLD).^69^ FTO also suppresses the PPARβ/δ and AMPK pathways, which disrupts the lipid utilization of skeletal muscles, reduces insulin secretion, and leads to diabetic hyperlipidemia.^69^ Wu et al found that down-regulation of FTO increases the methylation of AMPK mRNA, thereby negatively regulating lipid accumulation.^70^ FTO also regulates adipogenesis by regulating autophagy^71^ and mRNA alternative splicing.^72^^,^^73^ In addition, Yu et al found that FTO increases CD36 (cluster of differentiation 36) expression and suppresses the anti-inflammatory effects of high-density lipoproteins (HDLs).^74^ ALKBH5, another eraser, also participates in the regulation of lipid metabolism. Carboxylesterase 2 (CES2) plays important roles in lipid mobilization and chemosensitivity to irinotecan. In HepaRG and HepG2 cells, ALKBH5 knockdown decreases CES2 mRNA and protein levels, leading to increased lipid accumulation.^75^

Research has indicated that many ‘readers' are also involved in adipogenesis. Mitochondrial carrier homology 2 (MTCH2) can promote adipogenesis of preadipocytes in porcine muscles. Jiang et al reported that MTCH2 expression is higher in obese-type breed pigs than in lean-type breeds while showing higher m^6^A levels in its mRNA. They found that FTO or YTHDF1 can suppress or increase MTCH2 expression, respectively.^77^ YTHDF1 knockout enhances the expression of the HSD17B11 gene, which regulates the formation of lipid droplets in esophageal cancer cells.^76^ YTHDF2 was found to target m^6^A-modified JAK2 transcripts and promote its mRNA decay, inhibiting adipogenesis by weakening the JAK2-STAT3-C/EBPβ pathway.^66^ YTHDF2 also accelerates the mRNA decay of CCNA2 and CDK2 by recognizing their m^6^A-modified transcripts, which prolongs cell cycle progression and suppresses adipogenesis.^65^ In addition, YTHDF2 is involved in the degradation regulation of focal adhesion kinase family interacting protein of 200 kD (FIP200), a component of the ULK1 complex that participates in the initiation process of autophagy to regulate adipogenesis.^78^ ATG5 (autophagy related 5) and ATG7 were also reported to be targets of YTHDF2. FTO silencing-mediated higher m^6^A levels of ATG5/7 increase YTHDF2-mediated decay, thus decreasing autophagy and adipogenesis.^71^ Furthermore, YTHDC2 was found to be decreased in NAFLD patients and the livers of lean mice, and suppressing YTHDC2 promoted triglyceride (TG) accumulation. Mechanistically, YTHDC2 binds to some adipogenesis-related genes, including SREBP1c, FASN, and acetyl-coenzyme A carboxylase 1 (ACC1), leading to decreased mRNA stability and gene expression.^79^ ZFP217 can modulate m^6^A levels by increasing the transcription of FTO and then promote adipogenesis.^80^ Zhong et al reported that m^6^A is a bridge between lipid metabolism and the circadian clock; meanwhile, they also found that the knockdown of METTL3 and YTHDF2 impacts lipid metabolism by affecting PPARα transcription and translation.^81^ Guo et al reported that HNRNPA2B1 knockdown inhibits the expression of the fatty acid synthetic enzymes ATP citrate lyase (ACLY) and ACC1, which decrease lipid accumulation in esophageal cancer cells.^82^ The IGF2BP family has also been reported to promote adipogenesis, but the target mRNAs require further investigation.^83^

Research indicated that a high-fat diet increases the methylation level of lipid metabolic genes,^84^ and oxidized low-density lipoproteins (ox-LDL) reduce the m^6^A level in human endothelium and monocyte cells.^85^ These studies have shown that there is a regulatory loop between lipid metabolism and m^6^A regulation. In summary, m^6^A modification is widely involved in lipid metabolism, including adipocyte differentiation, *de novo* synthesis of lipids, FAO, and transduction of lipid-mediated signals.

## The function of m^6^A modification in carbohydrate metabolism

**Figure 4:**
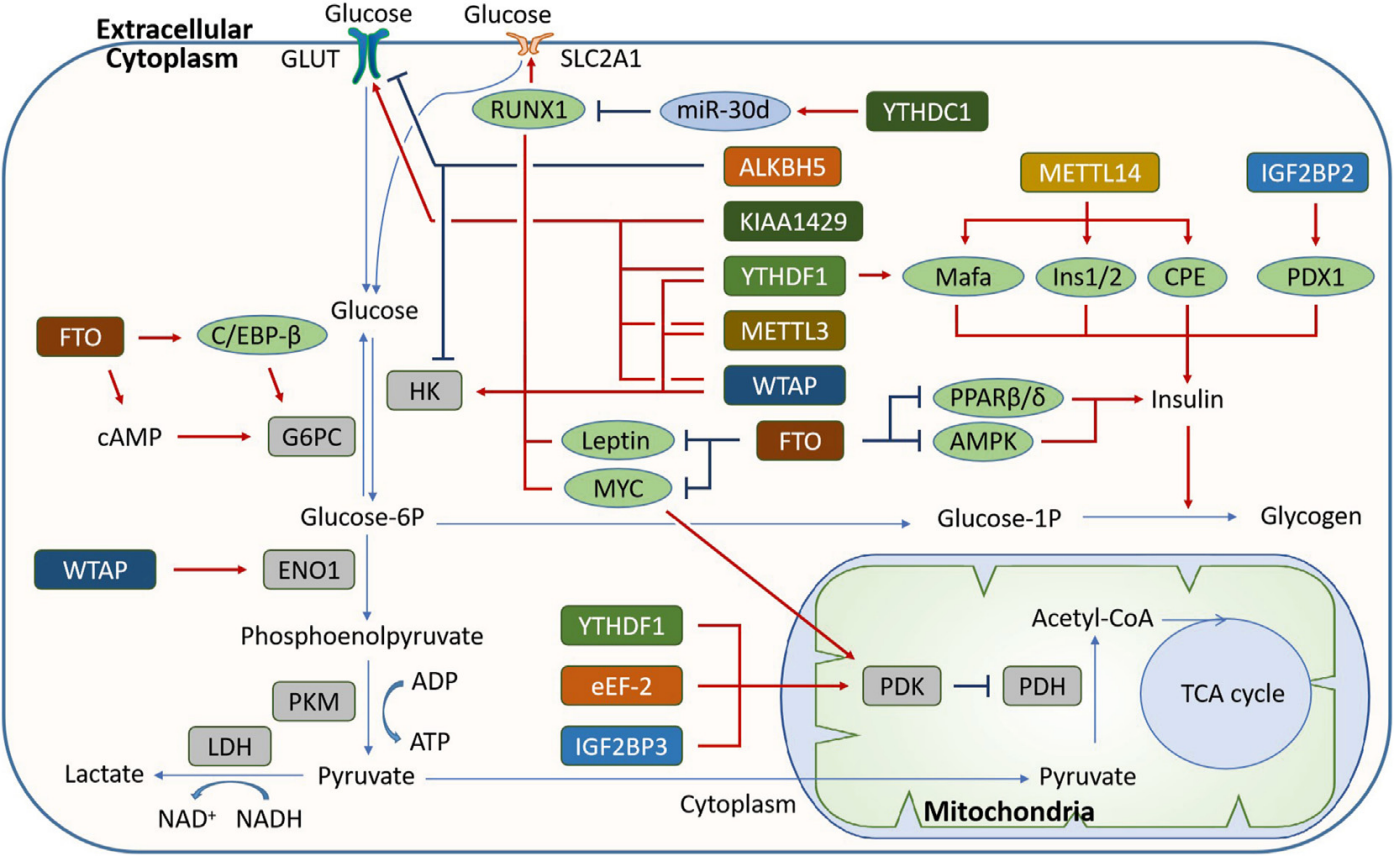
The function of m^6^A modification in carbohydrate metabolism. Carbohydrate metabolism is regulated by m^6^A modification at multiple levels including the synthesis and release of insulin, glycogen synthesis, glycolysis, and oxidative phosphorylation through regulating many carbohydrate metabolic genes.

Carbohydrates are another important source of energy and structural and signal substances, of which glucose is the most important. The homeostasis of glucose is closely related to energy requirements in physiological and pathological states. In most tissues, glucose eventually generates ATP through the Krebs cycle. In some cases, such as hypoxia or cells lacking mitochondria, glucose is decomposed into lactate, generating NAD^+^ and a small amount of ATP through anaerobic glycolysis. In the liver and muscles, excessive glucose is primarily changed into glycogen for glucose storage. In this section, we summarized the m^6^A modification in glucose metabolism (Fig. 4).

Insulin is a vital hormone for maintaining blood sugar balance and glucose metabolism. de Jesus et al reported that the m^6^A level of EndoC-βH1 cells from T2D patients is reduced significantly, and AKT phosphorylation and PDX1 expression are also decreased, which impairs insulin secretion.^86^ Shen et al reported that the m^6^A contents in RNA from T2D patients are significantly lower compared with the control groups and the lower m^6^A level in T2D may be associated with FTO instead of ALKBH5.^87^ In the β-cells of both T2D patients and a diabetic mouse model, decreased expression of METTL3/14 impairs the maturation of β-cells by decreasing the stability of MarfA mRNA, which leads to hyperglycemia and hypoinsulinemia.^88^ Liu et al also found that depletion of METTL14 leads to glucose intolerance and reduces insulin secretion by decreasing the expression of Ins, Ins2, and CPE.^89^ However, Xie et al reported that METTL3 is up-regulated in the liver tissue of T2D patients and high-fat diet-fed mice. Hepatocyte-specific deficiency of METTL3 enhances insulin sensitivity and suppresses fatty acid synthesis by decreasing the m^6^A level and expression of FASN mRNA.^60^ IGF2BP2 can directly bind to PDX1 mRNA, promote its translation, and lead to increased insulin secretion and β-cell proliferation.^90^ Besides, a high level of glucose enhances FTO mRNA expression but has no obvious effect on METTL3 and METTL14,^91^ which hints at the existence of a regulation loop.

Glucose metabolism-related processes can also be directly regulated by m^6^A.^92^ Studies have shown that many “writers” are involved in glucose metabolism. C5aR1-positive neutrophils can enhance the stability of WTAP by activating ERK1/2 signaling and thus increase m^6^A methylation of ENO1 mRNA to promote glycolysis.^93^ METTL3 and WTAP can target hexokinase 2 (HK2) mRNA and recruit YTHDF1 to enhance its mRNA stability.^94^^,^^95^ Shen et al reported that METTL3 interacts with GLUT1 and HK2 mRNA to increase their stability, which promotes glucose uptake and glycolysis in colorectal cancer.^96^ METTL3 was also reported to induce GLUT1 translation.^97^ In addition, KIAA1429 increases the m^6^A levels of the long noncoding RNA (lncRNA) Linc00958, thereby promoting the interaction of Linc00958 with GLUT1 mRNA to increase its mRNA stability.^98^ METTL14 knockdown enhances the mRNA stability of bromodomain PHD finger transcription factor (BPTF), leading to glycolytic reprogramming.^99^ The m^6^A “easers” are involved in carbohydrate metabolism processes. Studies have shown that FTO regulates mitochondrial function and the expression of many glucose metabolic genes, including phosphoenolpyruvate carboxykinase-mitochondrial (PEPCK-m) and glucose-6-phosphatase (G6PC).^100^^,^^101^ Huang et al found that FTO suppresses glycolysis by decreasing the stability of APOE mRNA in an m^6^A-dependent manner.^102^ The forkhead box protein O1 (FOXO1) is a transcription factor that regulates hepatic gluconeogenesis by increasing G6PC expression. Many research groups have reported that the mRNA expression level of FOXO1 is positively correlated with FTO and serum glucose.^91^^,^^103^ WNT/β-catenin increases the m^6^A level of MYC mRNA and promotes its translation by suppressing FTO expression, which promotes tumor cell glycolysis.^104^ LncRNAs proximal to the X-inactive specific transcript (JPX) decrease the m^6^A level and increase the stability of phosphoinositide-dependent kinase-1 (PDK1) mRNA by recruiting FTO to PDK1 mRNA, facilitating aerobic glycolysis in glioblastoma multiforme.^105^ FTO also increases the expression of cAMP-responsive element binding protein 1 (CREB1) and C/EBP-β to regulate gluconeogenesis.^100^ FTO overexpression in mouse liver decreases Y705 phosphorylation of STAT3, leading to increased G6P expression.^101^ Yu et al reported that ALKBH5 knockdown up-regulates the expression of casein kinase 2 α (CK2α), GLUT, HK1, and other glycolysis-related proteins.^106^ Many “readers” are also reported to be associated with the regulation of carbohydrate metabolism. The YTHDF1/eEF-2 complex and IGF2BP3 can enhance the mRNA stability and translation of pyruvate dehydrogenase kinase 4 (PDK4), a regulator in glycolysis and ATP generation.^107^ YTHDC1 enhances the maturation of miR-30 d in an m^6^A-dependent manner to inhibit aerobic glycolysis by targeting RUNX1, which binds to the promoters of HK1 and SLC2A1.^108^ LncRNA LINRIS promotes aerobic glycolysis in an m^6^A-mediated manner, and LINRIS knockdown decreases the downstream effects of IGF2BP2, especially MYC-induced glycolysis in colorectal cancer (CRC) cells.^108^

## The m^6^A modification in amino acid metabolism

Similar to carbohydrates and lipids, amino acids are multifunctional molecules that mainly act as the basic element of proteins. In mammals, amino acids are traditionally classified into essential and nonessential groups depending on whether they can be *de novo* synthesized *in vivo*. In this section, we will introduce the progress of m^6^A modification in amino acid-related metabolism.

Recently, a group calculated the m^6^A/A ratio by ultrahigh-pressure liquid chromatography coupled with triple-quadrupole tandem mass spectrometry (UHPLC-QQQ-MS/MS) in bacterial mRNA. Functional enrichment analysis showed that m^6^A peaks exist in many amino acid metabolism genes, including gabD (encodes succinate-semialdehyde dehydrogenase in *E. coli*), gabT (4-aminobutyrate aminotransferase in *E. coli*), and Idh (encodes leucine dehydrogenase in *P. aeruginosa*).^109^ Li et al reported that METTL14 may participate in the regulation of glutamic oxaloacetic transaminase 2 (GOT2), cysteine sulfonic acid decarboxylase (CSAD), and suppressor of cytokine signaling 2 (SOCS2) in hepatocellular carcinoma (HCC).^110^ Besides, METTL16 reprogrammes branched-chain amino acid (BCAA) metabolism by promoting the expression of BCAA transaminase 1 (BCAT1) and BCAT2, while depletion of METTL16 suppresses the initiation/development and stem cell self-renewal of acute myeloid leukemia (AML).^111^ Glutamate metabolism is an important metabolic process in cells that is always aberrant in cancer.^112^ Glutaminase (GA), encoded by the Gls gene (GLS), catalyzes the hydrolysis of glutamine to glutamate and ammonia. Research has indicated that YTHDF1 binds to the 3′UTR of GLS1 mRNA to enhance its translation in cisplatin-resistant CRC cells.^113^ These studies demonstrated that m^6^A modification is involved in the regulation of amino acid metabolism, but the mechanism requires further investigation.

## m^6^A modification regulates metabolism by regulating mitochondrial function

Mitochondria plays an important role in physiological processes, such as energy production, synthesis and decomposition of substances, apoptosis, and immunity. In this section, we will introduce the role of m^6^A in metabolism regulation by regulating mitochondrial function.

Peroxisome proliferator-activated receptor gamma coactivator 1-alpha (PGC-1α) is a master regulator of mitochondrial biogenesis.^114^ In inflammatory monocytes, METTL3 and YTHDF2 cooperatively suppress the expression of PGC-1α, and reduce ATP production and oxygen consumption rate (OCR), while METTL3 knockdown blocks oxLDL-induced inflammation damage of mitochondria.^115^ Docosahexaenoic acid (DHA) increases aerobic oxidation and mitochondrial biogenesis through increasing PGC-1α expression. Mechanistically, DHA enhances FTO expression, which reduces the m^6^A level and YTHDF2-mediated decay of DNA damage-induced transcript 4 (Ddit4) mRNA. Consequently, Ddit4 promotes PGC-1αexpression.^116^ In hematopoietic stem cells (HSCs), deficiency of IGF2BP2 increases mitochondrial activity by accelerating mRNA decay of Bmi1.^117^ Kang et al reported that FTO overexpression inhibits mitochondrial fission and promotes fusion to decrease its content and ATP levels through regulating multiple mitochondrial fission and fusion regulators.^67^ FTO enhances adipogenesis to inhibit mitochondrial unfolded protein response-induced apoptosis by activating the JAK2/STAT3 signaling pathway in adipocytes.^66^^,^^118^ FTO also regulates myogenic differentiation by affecting mitochondria biogenesis and function. FTO down-regulation decreases mitochondria mass, mitochondrial DNA content, PGC-1α expression, and ATP production by inhibiting mTORC1.^119^ Müller et al indicated that ALKBH1 can localize to mitochondria and affect the proliferation of HEK293 and HEK293T cells in different media, however, the mechanism requires further investigation.^120^

## The m^6^A modification in other metabolic pathways

In addition to the research described above, m^6^A modification has been proven to act as a master regulator in other metabolic pathways. In hypopharyngeal squamous cell carcinoma (HPSCC) patients, YTHDF1 increases the translation of TFRC to enhance iron metabolism.^121^ In protecting against pancreatic ductal adenocarcinoma (PDAC), overexpression of ALKBH5 reduces the intracellular iron level by regulating many iron-regulatory proteins, including F-box and leucine-rich repeat protein 5 (FBXL5), solute carrier family 25 member 28 (SLC25A28), and SLC25A37.^122^ Mosca et al found that B_12_ deficiency reduces SAM levels *in vitro* and *in vivo*, which may be caused by a wide decrease in m^6^A due to FTO up-regulation.^123^

TCA is the core pathway of multiple metabolic processes, including nucleic acids, carbohydrates, lipids, and amino acids. The metabolites of many substances supplement the TCA requirements, and the intermediates of TCA also provide a carbon skeleton, reduction equivalent, and ATP for the synthesis of these substances.^124^ For instance, α-ketoglutarate (α-KG) is the key intermediate of TCA and is also the carbon skeleton of glutamate and glutamine.^112^ Both FTO and ALKBH5, m^6^A erasers identified to date, are α-KG-dependent dioxygenases.^125^^,^^126^ These studies suggested that the metabolic process and m^6^A modification are mutually regulated processes. Metabolic progression is extremely complex, and multiple studies have indicated that m^6^A modification is widely involved in various metabolic processes. It is difficult to completely reveal the relationship between m^6^A modification and cellular metabolism; therefore, more research is needed.

## The role of m^6^A modification in metabolic diseases

Aberrant metabolism leads to many diseases called metabolic diseases, such as cancer, obesity, gout, cardiovascular disease, and type 2 diabetes (T2D).^127^, ^128^, ^129^, ^130^, ^131^ The metabolic process is extremely complex, and targeted therapy is challenging. We have summarized the function of m^6^A modification in different cellular metabolism processes, and in the following section, we will summarize the function of aberrant metabolism in different diseases (Table 4).

**Table 4:** The m^6^A mediated metabolic aberrance in metabolic disease.

Disease type	m6A regulator	Target gene	Metabolism type	Function	Reference
**HCC**	FTO	↑ FASN	Lipid	↓ Apoptosis	68
	METTL3	↓ SOCS2	Lipid	↑ Proliferation, migration, colony formation	137
		↑ LINC00958	Lipid	↑ Proliferation, metastasis	141
	METTL14	↑ CSAD, GOT2, SOCS2	Glucose, amino acids	↓ Proliferation, migration	110
	IGF2BP3	↑ PDK4	Glucose	↑ Proliferation	107
**ESCA**	HNRNPA2B1	↑ ACLY, ACC1	Lipid	↑ Proliferation, metastasis	82
	FTO	↑ HSD17B11	Lipid	↑ Proliferation, migration	76
**NSCLC**	METTL3	↑ ABHD11-AS1	Glucose	↑ Proliferation	138
**CRC**	YTHDF1	↑ GLS1	Amino acids	↑ Cisplatin resistance	113
	IGF2BP1	↑ RBRP	Glucose	↑ Proliferation, colony formation, metastasis	146
	IGF2BP2	↑ HK2, GLUT1	Glucose	↑ Proliferation, colony formation	96
		↑ MYC	Glucose	↑ Proliferation	140
		↑ ZFAS1	Glucose	↑ Proliferation, metastasis ↓ Apoptosis	139
**CC**	METTL3	↑ HK2	Glucose	↑ Proliferation	95
	YTHDF1	↑ PDK4	Glucose	↑ Proliferation	107
**GC**	KIAA1429	↑ GLUT1	Glucose	↑ Proliferation, metastasis	98
	WTAP	↑ HK2	Glucose	↑ Proliferation, metastasis	94
**PDAC**	YTHDC1	↓ miR-30 d	Glucose	↓ Proliferation, metastasis, angiogenesis	108
**BLCA**	ALKBH5	↓ CK2α	Glucose	↑ Apoptosis ↓ Proliferation, chemoresistance	106
**LUAD**	FTO	↓ MYC	Glucose	↓ Proliferation, metastasis	104
**Glioma**	IGF2BP2	↑ SHMT2	Amino acids	↑ Proliferation	142
**GBM**	FTO	↑ PDK1	Glucose	↑ Proliferation, TMZ resistance	105
**BC**	WTAP	↑ ENO1	Glucose	↑ Proliferation	93
	FTO	↑ PPARγ	Lipid	↑ Proliferation	186
**RCC**	METTL14	↓ BPTF	Glucose	↓ Metastasis	99
	FTO	↑ PGC-1α	Multiple types	↓ Proliferation	144
**AML**	METTL14	↑ MYC	Glucose	↑Proliferation	185
	IGF2BP2	↑ MYC, SLC1A5, GPT2	Amino acids	↑ Proliferation ↓ Apoptosis	147
**OSCC**	METTL3	↑ MYC	Glucose	↑ Proliferation, metastasis	180
**Obesity**	WTAP/METTL3/14 complex	↑ CCNA2	Lipid	↑ Adipogenic differentiation ↓ Insulin sensitivity	59
	METTL3	↓ JAK1	Lipid	↓ Adipogenic differentiation	54
		↑ FASN	Glucose	↓ Insulin sensitivity ↑ Adipogenesis	60
		↓ CCND1	Lipid	↓ Adipogenesis	52
		↑ PRDM16, PPARG,UCP1	Lipid	↑ Adipogenesis	55
	FTO	↑ CCNA2, CDK2	Lipid	↑ Adipogenesis	65
		↑ JAK2	Lipid	↑ Adipogenesis	66
		↑ RUNX1T1	Lipid	↑ Adipogenesis	73
		↑ ATG5/7	Lipid	↑ Adipogenesis	71
		↑ FASN, SCD1, MGAT1	Lipid	↑ Adipogenesis	67
	YTHDF2	↓ PPARα	Lipid	↓ Lipid accumulation	81
**T2D**	METTL3	↑ MafA	Glucose	↑ β-cells maturation	88
	METTL14	↑ PDX1	Glucose	↑ β-cells proliferation, insulin secretion	86
		↑ MafA	Glucose	↑ β-cells maturation	88
		↓sXBP-1, IRE1α	Glucose	↑ Insulin secretion	152
	IGF2BP2	↑ PDX1	Glucose	↑ β-cells maturation, insulin secretion	90

Note: ↑ means upregulation and ↓ means downregulation.

HCC: Hepatocellular cancer; ESCA: Esophageal cancer; NSCLC: Non-small cell lung cancer; CRC: colorectal cancer; CC: Cervical cancer; GC: Gastric cancer; PDAC: Pancreatic ductal adenocarcinoma; BLCA: Bladder cancer; LUAD: Lung adenocarcinoma; GBM: glioblastoma multiforme; BC: Breast cancer; RCC: Renal cell carcinoma; AML: acute myeloid leukemia; OSCC: oral squamous cell carcinoma; T2D: Type 2 diabetes.

## The role of m^6^A-mediated metabolism in cancer

**Figure 5:**
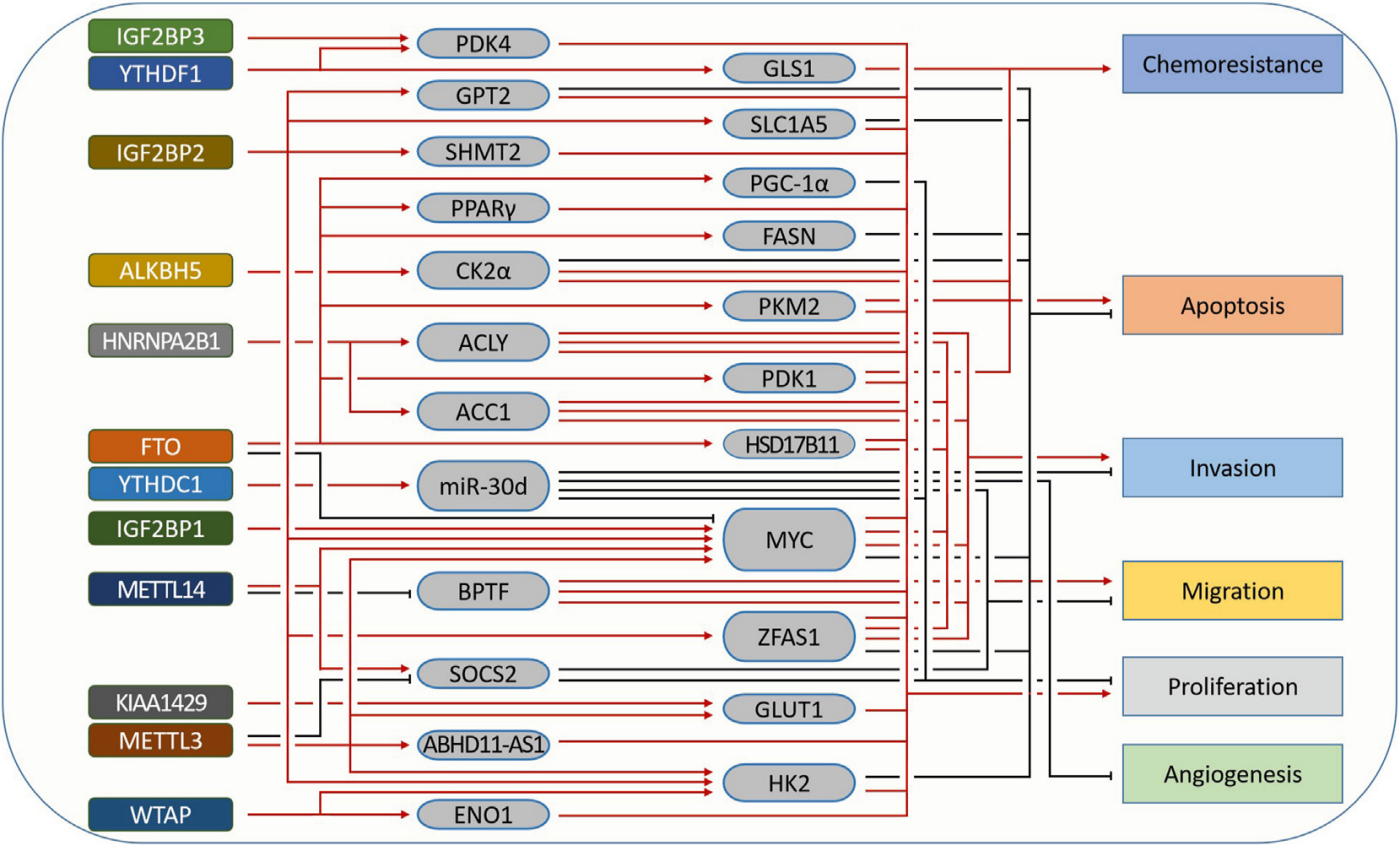
The m^6^A mediated metabolic aberrance in cancer. Cancer cells possess many malignant phenotypes, such as enhanced proliferation, migration, invasion, chemoresistance, angiogenesis, and resistance to apoptosis. Different m^6^A regulators act distinct roles in tumorigenesis and development by regulating different targets.

It is well known that tumorigenesis is a multifactorial matter, including the activation of oncogenes, gene mutations, inactivation and/or mutation of tumor suppressors, anti-apoptosis, and metabolic reprogramming.^132^, ^133^, ^134^ To meet the high demand for energy and materials, the metabolic pattern of tumor cells is usually different from that of normal cells. m^6^A modification has been proven to participate in the malignant progression of a tumor.^135^ In this section, we will introduce the role of m^6^A-mediated metabolic reprogramming in human cancer (Fig. 5).

Lipids are important structural, energy, and signaling molecules in cells. In MYC-overexpressing triple-negative breast cancer (TNBC) cells, inhibition of FAO decreases energy metabolism significantly.^136^ FTO can up-regulate PPARγ expression, enhance adipogenesis, and thus promote the proliferation of breast cancer cells.^186^ Chen et al found that m^6^A triggers the expression of CPT1B and ABCB1 by increasing ERRγ expression, which subsequently enhances the chemoresistance of tumor cells.^61^ However, increased *de novo* lipogenesis provides structural material for cancer cell proliferation. Sun et al reported that knockout of the m^6^A eraser FTO inhibits FASN expression, leading to reduced *de novo* lipogenesis and promoting apoptosis of HepG2 cells.^68^ METTL14 promotes SOCS2 expression to inhibit the progression of liver cancer,^110^ while METTL3 inhibits SOCS2 expression in a YTHDF2-dependent manner,^137^ but whether METTL3 regulates the metabolism of HCC through SOCS2 requires more direct evidence. In esophageal cancer, the m^6^A reader HNRNPA2B1 promotes the expression of ACLY and ACC1, which increases lipid accumulation.^82^

Aerobic glycolysis, also called the Warburg effect, is a common characteristic of glucose metabolism in most tumors.^132^ Xue et al found that METTL3 enhances the expression of ABHD11-AS1, which promotes the proliferation and Warburg effect of non-small cell lung cancer.^138^ In colorectal cancer, IGF2BP2 enhances the ZFAS1-OLA1 axis and promotes cell proliferation and the Warburg effect.^139^ LncRNA LINRIS can stabilize IGF2BP2 and promote aerobic glycolysis through the LINRIS/IGF2BP2/c-Myc axis.^140^ In cervical cancer, METTL3 promotes tumorigenesis and the Warburg effect by enhancing the stability of HK2 mRNA in a YTHDF1-dependent manner.^95^ WTAP also enhances the stability of HK2 mRNA in gastric cancer.^94^ In HCC, the expression of METTL3 and LinC00958 is positively related, which promotes cell proliferation and metastasis by enhancing lipogenesis.^141^ KIAA1429 also methylates and stabilizes LinC00958 to enhance aerobic glycolysis by promoting GLUT1 expression in gastric cancer.^98^ In pancreatic ductal adenocarcinoma, YTHDC1 increases the accumulation of miR-30 d, which suppresses RUNX1-induced expression of SLC2A1 and HK1, thereby inhibiting aerobic glycolysis.^108^ In bladder cancer, ALKBH5 reduces CK2α in an m^6^A-dependent manner, which inhibits glucose uptake and sensitizes tumor cells to cisplatin.^106^ In lung adenocarci-noma, wnt/β-catenin signaling inhibits FTO expression to promote glycolysis and tumorigenesis by increasing the m^6^A modification of c-Myc mRNA.^104^ In AML, METTL14 can promote the proliferation of cancer cells by increasing the expression of MYC.^185^ In multiple myeloma, FTO decreases the m^6^A level of WNT7B and then increases its expression, thus activating the Wnt pathway.^192^ Streptozotocin-treated astrocytes show higher levels of YTHDF1 and FTO, and inhibition of FTO sensitizes astrocytes to streptozotocin and elevates mitochondrial dysfunction.^143^ Besides, lncRNA JPX stabilizes PDK1 mRNA by enhancing FTO-mediated demethylation of PDK1 mRNA, thereby promoting aerobic glycolysis and temozolomide resistance of glioblastoma multiforme cells.^105^ PDK4 is a key regulator of glycolysis and ATP generation. In cervical and liver cancer, the YTHDF1/eEF-2 complex and IGF2BP3 bind to the m^6^A-modified 5′UTR of PDK4, which enhances its translation and mRNA stability, respectively.^107^ In breast cancer, C5aR1-positive neutrophils promote glycolysis and tumor progression by enhancing ENO1 expression in a WTAP-dependent manner.^93^ In clear cell renal cell carcinoma (ccRCC), Zhuang et al indicated that low expression of FTO correlates with poor prognosis, and FTO increases ROS production and impairs tumor growth by increasing expression of PGC-1α.^144^ Additionally, METTL14 deficiency decreases the m^6^A modification and increases the stability of BPTF mRNA, which further leads to glycolytic reprogramming and lung metastasis of RCC cells.^99^ ^18^F-FDG is an indicator of glucose uptake. Shen et al found that METTL3 increases ^18^F-FDG uptake by stabilizing HK2 and GLUT1 mRNA in an IGF2BP2/3-dependent manner, which subsequently enhances glycolysis in CRC.^96^

To sustain a proliferative drive, cancer cells require large amounts of amino acids.^112^ Studies have shown that dysregulation of amino acid metabolism is implicated in cancer cell growth and that glutamine decomposition is one of the essential features of tumor energy metabolism.^124^^,^^145^ Serine hydroxymethyltransferase 2 (SHMT2) can catalyze the conversion of serine to glycine and one-carbon transfer reactions in mitochondria. Han et al reported that HOXA transcript antisense RNA, myeloid-specific 1 (HOTAIRM1) can bind to IGF2BP2 to maintain the stability of SHMT2 mRNA, and thus promotes glioma growth.^142^ Kan reported that glutamine is involved in energy generation and signal transmission in cancer cells by providing carbon and nitrogen.^112^ In colorectal cancer, up-regulated YTHDF1 decreases the cisplatin sensitivity of cancer cells by increasing the translation of glutaminase GLS1, and inhibition of GLS1 increases the therapeutic effect of cisplatin.^113^ LncRNA Linc00266-1 encodes a 71-amino acid peptide, named RNA binding regulatory peptide (RBRP). IGF2BP1 can bind to RBRP to increase c-Myc expression, thereby promoting tumorigenesis.^146^ In addition, the high expression of IGF2BP2 is related to the maintenance of HSCs.^117^ IGF2BP2 promotes AML development and self-renewal of stem/initiation cells through increasing the expression of MYC, SLC1A5, and GPT2 which are related to the glutamine metabolism pathway.^147^

## The role of m^6^A-mediated metabolism in obesity

**Figure 6:**
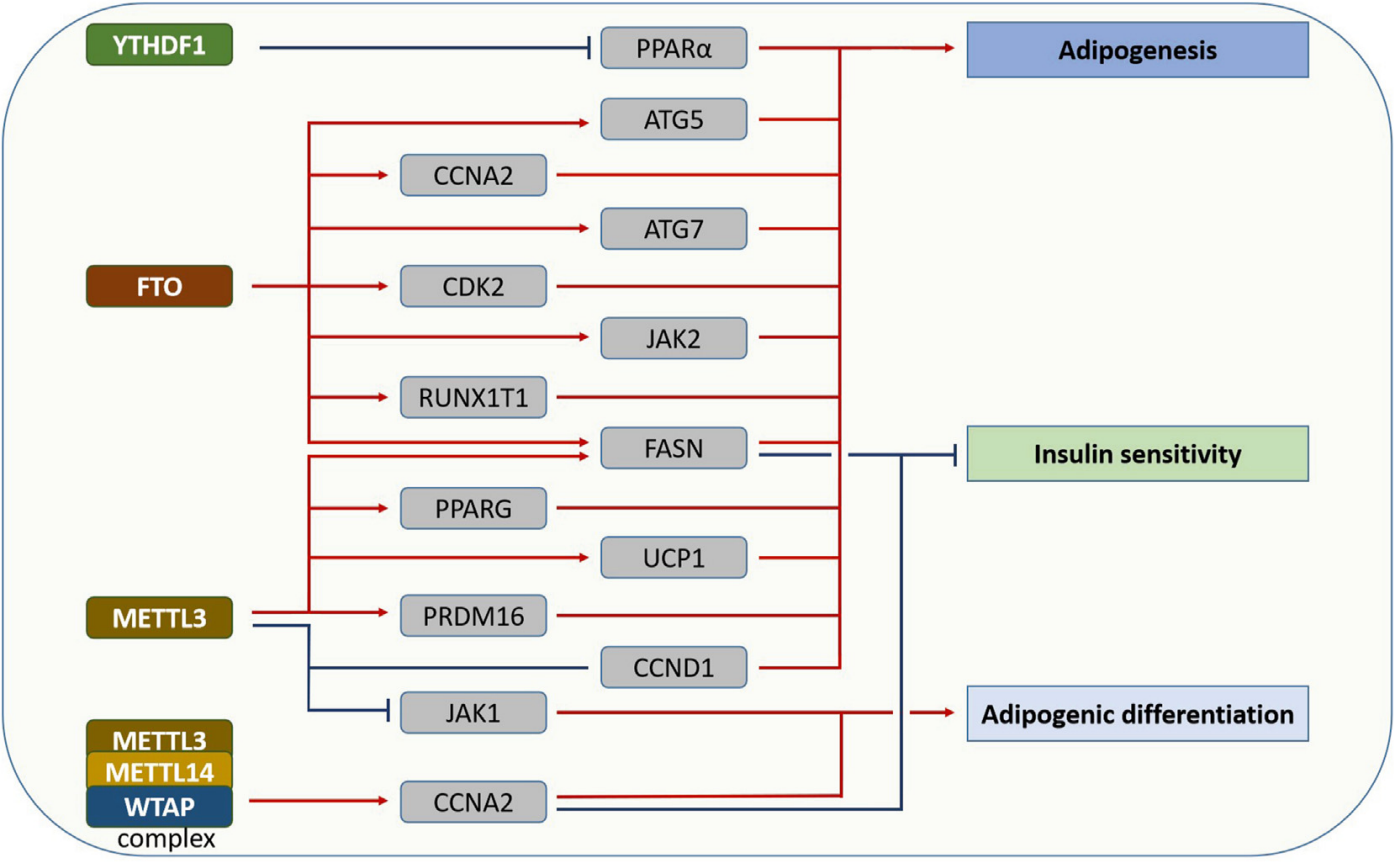
The m^6^A mediated metabolic aberrance in obesity. Obesity is a threat to human health and is the inducer of many diseases. m^6^A modification is a master regulator of obesity progress by regulating many metabolism pathways.

Obesity is a chronic metabolic disease that manifests as the excessive accumulation of fat and acts as an inducer of multiple diseases. Fat tissue can be divided into white adipose tissue and brown adipose tissue, which convert excess energy into lipid droplets or generate heat, respecti-vely.^148^^,^^149^ Generally, obesity is the result of dysregulation of energy metabolism, characterized as excess energy being converted into lipid droplets and accumulation in adipose tissue.^150^ In this section, we will introduce the regulatory role of m^6^A modification in obesity-associated processes (Fig. 6).

FTO was first found to be associated with human obesity in 2007,^62^ and subsequently, its demethylation effect was discovered in 2011, which aroused great interest from researchers in the role of m^6^A in obesity. Karra et al reported that the rs9939609A allele of FTO enhances its expression and subsequently increases ghrelin expression.^151^ Recent studies have shown that FTO participates in and promotes adipogenesis through several mechanisms, including regulating mitotic clonal expansion and autophagy.^59^^,^^65^^,^^66^^,^^71^, ^72^, ^73^ FTO was also found to play a regulatory role in lipid metabolism. Kang et al reported that FTO decreases mitochondrial content and promotes TG deposition in HepG2 cells.^67^ Like FTO, METTL3 also participates in both adipogenesis and lipid metabolism. Interestingly, several studies have shown that METTL3 can both promote and inhibit adipogenesis. On the one hand, Kobayashi et al reported that METTL3, METTL14, and WTAP positively control adipogenesis by promoting cell cycle translation in mitotic clonal expansion and affecting insulin sensitivity.^59^ ZFP217 knockdown inhibits adipogenesis by enhancing METTL3-induced expression of cyclin D1^52^. On the other hand, Yao et al found that METTL3 inhibits BMSC adipogenic differentiation by targeting the JAK1/STAT5/C/EBPβ pathway in a YTHDF2-dependent manner.^54^ Wang et al found that METTL3 is essential for the postnatal development of brown adipose tissue in mice, and deletion of METTL3 decreases the expression of Prdm 16, Pparg, and UCP1 and impairs the maturation of brown adipose tissue.^55^ Moreover, hepatocyte-specific deficiency of METTL3 enhances insulin sensitivity and suppresses fatty acid synthesis by decreasing the m^6^A level and expression of FASN mRNA.^60^ YTHs were also reported to regulate adipogenesis and lipid metabolism. YTHDF1 and YTHDF2 can recognize m^6^A-bound mRNA and then promote its translation or degradation. Zhong et al reported YTHDF2 knockdown increases the transcription and translation of PPaRα and then increases lipid accumulation in HepG2 cell.^81^

## The role of m^6^A-mediated metabolism in T2D

Type 2 diabetes (T2D) is a complicated metabolic disease caused by many factors, including insulin deficiency and insulin resistance. T2D can lead to serious complications, such as cardiovascular diseases and diabetic ketoacidosis. To date, several studies have shown that m^6^A modification regulates the development of T2D. In this section, we will introduce the regulatory role of m^6^A modification in T2D.

Jesus et al reported that several T2D-related transcripts involved in cell cycle progression, insulin secretion, and the insulin/IGF1-AKT-PDX1 pathway were hypomethylated in T2D islets compared with normal controls.^86^ β-cell-specific METTL14 knockout mice display reduced m^6^A levels, β-cell proliferation, and insulin degranulation, which is consistent with the islet phenotype of early-onset human T2D and mortality.^86^ Men et al also reported that METTL14 knockout in β-cells activates the IRE1α/sXBP-1 pathway and then causes glucose intolerance and reduces insulin secretion.^152^ Similarly, Wang et al found that the expression of METTL3/14 is down-regulated in the β-cells of both a diabetic mouse model and T2D patients.^88^ In addition, mice with specific knockout of METTL3/14 in Ngn3^+^ endocrine progenitors develop hyperglycemia and hypoinsulinemia. Their investigation demonstrated that METTL3/14 increase the mRNA stability of musculoaponeurotic fibrosarcoma oncogene family A (MafA) to regulate maturation and mass expansion but differentiation of neonatal β-cells.^88^ Regué et al found that IGF2BP2 directly binds to m^6^A-modified PDX1 mRNA to increase its translation, which subsequently enhances the proliferation and insulin secretion of pancreatic β-cells.^90^ Interestingly, Xie et al reported that the m^6^A-modified RNA level and METTL3 are up-regulated in T2D patient liver tissues, positively correlated with insulin resistance, and negatively correlated with β-cell function.^60^ Moreover, aberrant glucose and m^6^A modification may be a positive feedback loop in the progression of T2D. Kobayashi et al reported that WTAP heterozygous mice have a higher insulin sensitivity and are insusceptible to diet-induced obesity.^59^ Yang et al found that high glucose enhances FTO expression and is accompanied by increased expression of FOXO1, G6PC, and diacylglycerol O-acyltransferase 2 (DGAT2), which are associated with serum glucose.^91^

## The role of m^6^A in mediating metabolism in other diseases

In addition to the diseases mentioned above, m^6^A modification was also found to be closely related to other human diseases, such as neuronal disorders and cardiovascular diseases. Richard et al found that METTL5 is enriched in the nucleus and synapses of human hippocampal neurons and that its biallelic variants lead to intellectual disability and microcephaly.^153^ Han et al reported that m^6^A levels are positively related to the development of Alzheimer's disease.^154^ In Parkinson's disease, the m^6^A level is also decreased, which accounts for the high expression of N-methyl-d-aspartate (NMDA) receptor 1 and subsequent oxidative stress and Ca^2+^ influx-induced apoptosis of dopaminergic neurons.^155^ Engel et al found that depletion of FTO and METTL3 in adult neurons increased fear memory, and m^6^A was impaired in major depressive disorder.^156^

There are increasing studies demonstrating that m^6^A is associated with the occurrence and development of cardiovascular diseases.^157^ Dorn et al found METTL3 overexpression in cardiomyocytes can cause hypermethylation of mitogen-activated protein kinase kinase 6 (MAP3K6), MAP4K5, and MAPK14, activate them, and induce cardiac hypertrophy.^158^ Gao et al reported that the CHAPIR-PIWIL4 complex binds to METTL3, blocks its activity, and then up-regulates PARP10 expression. The increased PARP10 inhibits the kinase activity of GSK3β, leading to the accumulation of NFATC4 and pathological hypertrophy.^159^

## Therapeutic strategy based on m^6^A modification

As mentioned above, numerous studies have indicated that m^6^A modification is a crucial regulator of metabolic processes. m^6^A dysregulation is accountable for many diseases, which provides a new direction for the treatment of metabolic diseases. At present, many m^6^A-targeting inhibitors have been found, and some of them show satisfactory application prospects. In this section, we summarized the current m^6^A-targeted compounds and their prospects in treating metabolic diseases (Table 5).

**Table 5:** The identified m^6^A-targeted compounds.

Inhibitor	Full name	Structure	Target gene	Mechanism	Application	Reference
Quercetin	Quercetin		Methyltransferase	Decreases METTL3 expression	Cervical cancer cells	176
3-DAA	3-deazaadenosine		Methyltransferase	Interrupts methyl insertion m6A into mRNA	Chick embryo cells and Rous sarcoma	162
STM2457	STM2457		METTL13	Competitively binds to METTL13 active site	AML and iCCA cells	177,178,182
UZH1a	UZH1a		METTL3	Competitively binds to METTL13 active site	MOLM-13	179
SAH	S-adenosylhomocysteine		METTL3/14	Competitively binds to active site	HEK293 cells	160,161
Eltrombopag	Eltrombopag		METTL3-METTL14 complex	Directly binds to enzyme protein	MOLM-13	184
D2-HG	D2-hydorxyglutarate		FTO	Disrupts α-KG- dependent dioxygenases	Diffuse large B-cell lymphoma	164
Rhein	Rhein		FTO	Competitively binds to FTO active site	AML and SK-N-BE cell	165,191
MA	Meclofenamic acid		FTO	Recognizes nucleotide recognition lid	Hela cells	166
MA2	Meclofenamic acid 2		FTO	Competitively binds to FTO active site	Glioblastoma stem cells	196
MO-I-500	MO-I-500		FTO	Competitively binds to FTO active site	TNBC	187
Compound 12	Compound 12		FTO	Competitively binds to FTO active site	Hela cells	169
IOX3	FG-2216		FTO	Competitively binds to FTO active site	C2C12 cells	167,168
R-2HG	R-2-hydroxyglutarate		FTO	Competitively binds to FTO active site	Leukemic cells, glioma	48
FB23	FB23		FTO	Competitively binds to FTO active site	AML	188
FB23-2	FB23-2		FTO	Competitively binds to FTO active site	AML	188
18,077	18,077		FTO	Competitively binds to FTO active site	Breast cancer cells	186
18,097	18,097		FTO	Competitively binds to FTO active site	Breast cancer cells	186
Entacapone	Entacapone		FTO	Competitively binds to FTO active site	Hep-G2 cells	103
EGCG	Epigallocatechin gallate		FTO	Decreases FTO expression	3T3-L1 cells	203
CS1	CS1		FTO	Binds tightly to FTO protein and blocks its catalytic pocket	AML	190
CS2	CS2		FTO	Binds tightly to FTO protein and blocks its catalytic pocket	AML	190
13a	13a		FTO	Competitively binds to FTO active site	AML	189
Ena15	Ena15		FTO	Competitively or uncompetitively binds to FTO active site	Glioblastoma cells	199
Ena21	Ena21		FTO	Competitively or uncompetitively binds to FTO active site	Glioblastoma cells	199
Curcumin	Curcumin		ALKBH5	Decreases ALKHB5 expression	3T3-L1 cells	204
MV1035	MV1035		ALKBH5	Competitively binds to active site	Glioblastoma cells	200
Tegaserod	Tegaserod		YTHDF1	Blocks the direct binding of YTHDF1 with mRNA	AML	193
BTYNB	2-{[(5-bromo-2-thienyl)methylene]amino} benzamide		IGF2BP1	Competitively binds to active site	iCCA and Ovarian cancer cells	197,198
CWI1-2	CWI1-2		IGF2BP2	Competitively binds to active site	AML	147
JX5	JX5		IGF2BP2	Competitively binds to active site	T-ALL cells	194

Up to now, there are many m^6^A-targeted compounds have been reported. S-adenosylhomocysteine (SAH), a methyl derivative of SAM, was reported to inhibit SAM-dependent methyltransferases by competing with adenosylmethionine.^160^ It binds to the catalytic site of METTL3/14 complex.^161^ 3-deazaadenosine (3-DAA), a SAH hydrolysis inhibitor, was proven to inhibit m^6^A by interrupting the insertion of m^6^A into mRNA.^162^ D2-hydroxyglutarate (D2-HG), an analog of α-ketoglutarate (α-KG), can disrupt α-KG-dependent dioxygenases and thus inhibit the activity of FTO.^163^^,^^164^ Rhein, a natural product from medicinal herbs, such as Rheum palmatum L, was proven to bind to the FTO active site and competitively prevent the recognition of m^6^A substrates, inhibiting FTO-mediated m^6^A demethylation.^165^ FG-2216 (IOX3), a known inhibitor of hypoxia-inducible factor prolyl-hydroxylases (PHDs), was proven to bind the 2-oxoglutarate and nucleotide binding sites of FTO to inhibit its enzyme activity.^167^^,^^168^ Compound 12 can occupy an unexplored substrate binding site and be demonstrated distinct selectivity for FTO against other AlkB subfamilies.^169^ Additionally, fluorescein derivatives can both inhibit FTO demethylation and label FTO proteins.^170^ Moreover, Simona et al found several unnamed small-molecule compounds that act as activators of the METTL3-METTL14-WTAP complex in HEK293 cells.^171^

Multiple studies have indicated that targeting m^6^A regulators is a promising strategy to treat some cancers. For instance, m^6^A modification improves the stability of circMDK to promote tumorigenesis in HCC.^172^ METTL3-depleted pancreatic cancer cells are more sensitive to cisplatin and gemcitabine.^173^ IGF2BP2 deficiency induces quiescence loss and impairs HSC function.^117^ The m^6^A level of osteosarcoma is positively associated with chemoresistance and poor prognosis.^174^ Many m^6^A target compounds have shown anticancer effects. Quercetin, a flavonol-type compound, can inhibit METTL3 expression and the proliferation of MIA PaCa-2 and Huh7 cells.^175^ Quercetin also inhibits the proliferation and invasion of HeLa and SiHa cells.^176^ UZH1a and STM2457, which inhibit METTL3 expression, can decrease the m^6^A level and inhibit the progression of AML cells.^177^, ^178^, ^179^ STM2457 also suppresses the tumor progression of oral squamous cell carcinoma cells,^180^ SHH subgroup medulloblastoma,^181^ and intrahepatic cholangiocarcinoma.^182^ Additionally, STM2457 enhances the anti-PD1 therapy effect of cervical squamous cell carcinoma.^183^ Eltrombopag, an allosteric inhibitor of the METTL3-14 complex, decreases the m^6^A levels and displays anti-proliferative effects in MOLM-13 cells.^184^ SPI1 directly decreases METTL14 expression in malignant hematopoietic cells.^185^ The compound 18,077 and 18,097, two selective inhibitors of FTO, can inhibit the cell cycle process of breast cancer.^186^ MO-I-500 also inhibits the survival and colony formation of breast cancer cells by inhibiting FTO.^187^ Besides, R-2HG,^48^ FB23/FB23-2,^188^ 13a,^189^ CS1/CS2,^190^ and Rhein^191^ can inhibit the progression of AML cells by inhibiting FTO activity. Tegaserod, a YTHDF1 inhibitor,^193^ and CWI1-2, an IGF2BP2 inhibitor,^147^ also show anti-leukemia effects *in vivo* and *in vitro*. JX5, another IGF2BP2 inhibitor, suppresses the activation of NOTCH1 and the growth of T-cell acute lymphoblastic leukemia.^194^ Meclofenamic acid (MA), a nonsteroidal anti-inflammatory drug, inhibits FTO activity by competing with FTO for binding to reduce the binding of m^6^A-containing RNA directly.^166^ In malignant lung cells, 3-DAA enhances lung cancer cell proliferation and migration through decreasing ZNRD1-AS1 expression in a YTHDC2-dependent manner, but MA inhibits this progress.^195^ MA2, the ethyl-ester derivative of MA, was found to suppress tumorigenesis in glioblastoma stem cells.^166^ The MA2-treated glioblastoma stem cell-grafted mice show decreased tumorigenesis and a preferable prognosis.^196^ BTYNB, an IGF2BP1 inhibitor, inhibits melanoma and ovarian cancer cell proliferation by suppressing c-Myc signalling.^197^ BTYNB also shows anti-tumor efficacy in a PDX model of intrahepatic cholangiocarcinoma.^198^ Ena15 and Ena21 are two novel ALKBH5 inhibitors, which inhibit the proliferation of glioblastoma multiforme cells.^199^ Besides, MV1035, another inhibitor of ALKBH5, can reduce the migration and invasion of U87 cells.^200^ In CRC and melanoma, ALK-04, an inhibitor of ALKBH5, can decrease the infiltration of immunosuppressive cells in the tumor microenvironment and suppress tumor growth.^201^

In addition to cancer, an increasing number of studies have focused on the treatment of obesity and other metabolic diseases by targeting m^6^A modification. Entacapone, a drug for the treatment of Parkinson's disease,^202^ was found to inhibit FTO activity and affect lipid and glucose metabolism.^103^ Epigallocatechin gallate, an extract from green tea, was discovered to target FTO and then inhibit adipogenesis, exhibiting an anti-obesity effect.^203^ Curcumin, a natural phenolic compound that shows an anti-obesity effect, has been reported to reduce the expression of ALKHB5 and then increase the translation of TNF receptor-associated factor 4 (TRAF4) by up-regulating its mRNA m^6^A modification level.^204^

## Conclusions and perspectives

As one of the major internal modifications in eukaryotic RNAs, the classic processes of m^6^A modification mainly involve methyltransferases, methylases, and m^6^A binding proteins, which are also called ‘writers’, ‘erasers’, and ‘readers’, respectively. m^6^A modification has been shown to play an essential role in regulating RNA processing, maturation, translation, and metabolism, and it also exerts critical functions in modulating cellular metabolism, development,^205^ and disease processes. RNA m^6^A modification has become a hot topic, and its role in cellular metabolism has been researched extensively in recent years. It is well known that m^6^A modification is essential in numerous cellular metabolic processes, which are important for maintaining the physiologic state. Mechanistically, m^6^A modification can regulate the expression and/or activity of various metabolic enzymes directly or indirectly. However, aberrant m^6^A modification is associated with the occurrence and development of multiple metabolic diseases, such as cancer, obesity, cardiovascular disease, and T2D.

As research has progressed, m^6^A-targeting drugs have provided new therapeutic directions for metabolic diseases. Some natural products from traditional medicine have been reported to possess m^6^A-targeting activity, such as rhein, curcumin, quercetin, and betaine.^176^^,^^191^^,^^204^^,^^206^ In addition, synthetic m^6^A-targeted drugs also show great potential in metabolic diseases such as FB23, FB23-2, and 18,097.^186^^,^^188^ Many studies have demonstrated that some m^6^A-targeting molecules alleviate a variety of diseases *in vitro* and in animal models. For example, 18,097 can suppress lung colonization of breast cancer cells. Mechanistically, 18,097 alters the m^6^A level of SOCS1 mRNA and subsequently activates the P53 signaling pathway.^186^ Curcumin shows a protective effect on metabolic diseases such as obesity. It reduces ALKBH5 to increase the expression of TRAF4, which promotes the degradation of PPARγ and thus inhibits adipogenesis.^204^ Moreover, m^6^A-targeted therapy may sensitize cancer cells to radiotherapy. Taketo et al found that METTL3-depleted pancreatic cancer cells are more sensitive to irradiation.^172^ However, there is still a long way t the application of the current m^6^A-targeting compounds in the clinical treatment of these diseases. Hopefully, the AI-assisted techniques in drug design, discovery, and development have quickly developed, which makes it more efficient, safer, and less costly to find effective medicine for multiple diseases.^207^

Although the intimate connection between m^6^A modification and cellular metabolism has been well-proven in many studies, research on the specific mechanism is still superficial. In this context, we introduced the identified roles of m^6^A modification in cellular metabolism and summarized the mechanism of aberrant m^6^A modification leading to metabolic diseases, expecting to provide some help for further investigation of m^6^A modification and cellular metabolism.

## Author contributions

Chen Yang and Xiao Yufeng conceived and designed this work. Xie Xia revised the manuscript. Hu Haiming collected most of the material and drafted the first three and last sections of this manuscript. Li Zhibin drafted the fourth and fifth sections of this manuscript and is the main figure and table maker. Liao Qiushi, Hu Yiyang, Gong Chunli, Gao Nannan, and Yang Huan helped a lot in the collection of materials and beautification of figures. All authors read and approved the final manuscript.

## Conflict of interests

The authors declare that there is no potential competing interest.

## Funding

This work was supported by the National Natural Science Foundation of China (No. 81874196), the Natural Science Foundation of Chongqing, China (No. cstc2021jcyj-msxmX0536), and the Undergraduate Scientific Research Cultivation Program of Army Medical University, Chongqing, China (No. 2021XBK22).

## Data availability

All the data of this study were available from the corresponding authors upon reasonable request.
